# Bioactive Peptides from Corn (*Zea mays* L.) with the Potential to Decrease the Risk of Developing Non-Communicable Chronic Diseases: In Silico Evaluation

**DOI:** 10.3390/biology13100772

**Published:** 2024-09-27

**Authors:** Caroline Cagnin, Bianca de Fátima Garcia, Thais de Souza Rocha, Sandra Helena Prudencio

**Affiliations:** Department of Food Science and Technology, State University of Londrina, Celso Garcia Cid Road, PR-445, Km 380, University Campus, Londrina 86057-970, PR, Brazil; carolinecagnin@gmail.com (C.C.); biancagarcia6@gmail.com (B.d.F.G.); tsrocha@uel.br (T.d.S.R.)

**Keywords:** antioxidant, angiotensin-converting enzyme, biological activities, DPP-IV, DPP-III, zein

## Abstract

**Simple Summary:**

The protein α-zein from corn has been studied both in vivo and in vitro for its potential to target treatments for non-communicable chronic diseases. This biological activity is achieved through the hydrolysis of the protein, which produces bioactive peptides. The study involved scientific research and a literature review to gather evidence of the biological activity of corn peptides. Additionally, databases and bioinformatics tools were utilized to simulate the enzymatic digestion of α-zein and confirm the bioactivity of the resulting peptides. The study discovered that the primary bioactivity is the inhibition of ACE, followed by the inhibition of DPP-IV and DPP-III, which are targets for treating hypertension and type-2 diabetes. In conclusion, conducting an in silico evaluation before proceeding to in vitro or in vivo studies can be efficient and cost-effective and contribute to better usage of corn gluten meals.

**Abstract:**

Studies have shown that corn (*Zea mays* L.) proteins, mainly α-zein, have the potential to act on therapeutic targets related to non-communicable chronic diseases, such as high blood pressure and type 2 diabetes. Enzymatic hydrolysis of proteins present in foods can result in a great diversity of peptides with different structures and possible bioactivities. A review of recent scientific research papers was performed to show evidence of the bioactive properties of corn peptides by in vitro assays. The α-zein amino acid sequences were identified in the UniProtKB protein database and then analyzed in the BIOPEP database to simulate enzymatic digestion and verify the potential biological action of the resulting peptides. The peptides found in the BIOPEP database were categorized according to the probability of presenting biological action using the PeptideRanker database. The aim was to use existing data to identify in silico the potential for obtaining biologically active peptides from α-zein, the main storage protein of corn. The analysis showed that the majority of peptide fragments were related to the inhibition of angiotensin-converting enzyme, followed by the inhibition of dipeptidyl peptidase IV and dipeptidyl peptidase III. Many drugs used to treat high blood pressure and type 2 diabetes work by inhibiting these enzymes, suggesting that corn peptides could be potential alternative agents. In vitro studies found that the primary bioactivity observed was antioxidative action. Both in vitro and in silico approaches are valuable for evaluating the bioactive properties resulting from protein hydrolysis, such as those found in α-zein. However, conducting in vitro studies based on prior in silico evaluation can be more efficient and cost-effective.

## 1. Introduction

Proteins are molecules composed of amino acids linked by peptide bonds. Proteins can play physiological roles related to health maintenance, transmission of nerve impulses, muscle formation, hormonal actions, etc. [1]. Food proteins have various properties from a food technology perspective. These include water and oil absorption and binding, gelation, emulsification, and foaming, among others. These properties depend on different mechanisms and molecular characteristics that may be intrinsic or extrinsic to the molecule itself. Extrinsic factors that affect protein functionality include pH, temperature, humidity, chemical additives, mechanical processing, ionic strength, and the presence of enzymes. Intrinsic characteristics include molecular size; shape; amino acid composition and sequence; net charge and distribution of charges; hydrophobicity/hydrophilicity ratio; secondary, tertiary, and quaternary structures; molecular flexibility/rigidity; and ability to interact with other components [1].

Food proteins also have different roles in human health. Processes such as gastrointestinal digestion and enzymatic, chemical, and microbiological hydrolysis can break peptide bonds, releasing fragments called peptides. Biologically active peptides are defined as inactive fragments of amino acid sequences in a protein that exhibit bioactivity after hydrolysis and interaction with an appropriate receptor in the human body [2].

Peptides naturally present in corn (*Zea mays* L.) or isolated from corn gluten or zein are safe for consumption, can be easily absorbed, and have low molecular mass (<6 kDa) [3]. Numerous investigations reported that corn proteins possess bioactive properties, including antioxidant [4], antihypertensive [5], hepatoprotective [6], anti-inflammatory [7], anti-cancer [8], antimicrobial [9], and dipeptidyl peptidase (DPP) IV (EC 3.4.14.5) inhibitory activities [10].

Cereals that may help prevent non-communicable chronic diseases, such as corn, are an important part of a healthy diet. According to the World Health Organization [11], non-communicable diseases such as cardiovascular diseases, chronic respiratory diseases, cancers, and diabetes are responsible for 41 million deaths annually worldwide, which accounts for 74% of all global deaths. Diabetes and hypertension are highly associated and are considered global public health problems because of their high incidence. Estimates from the International Diabetes Federation [12] indicate that there are 537 million people with diabetes worldwide, the most common being type 2 diabetes mellitus, which accounts for about 90% of cases. As for hypertension, the incidence is even higher, affecting 1.28 billion people in 2023 [12].

Biologically active peptides isolated from natural sources may help prevent chronic diseases and reduce adverse drug actions, in addition to contributing to the early treatment of diabetes and hypertension. Amino acid sequences in peptides that are responsible for specific biological responses can be predicted by in silico tools, reducing the cost and time required for the development of novel compounds [13]. Recently, databases such as UniProtKB [14] and BIOPEP-UWM [15] have been used to evaluate possible amino acid sequences in peptides and predict bioactivity [16].

The objective of this review was to collect information on biological activities associated with zein (prolamin) corn storage protein over the past 10 years from databases such as Scopus, Web of Science, Wiley, and Medline/PubMed. Additionally, the review aimed to compare these biological activities with those predicted using data from UniProtKB, BIOPEP-UWM, and PeptideRanker databases in order to identify potentially active peptides in the corn storage protein through an in silico approach. To the best of our knowledge, the specific assessment of the proteins focusing on their potential biological activities and the effects of simulated digestion using subtilisin and gastrointestinal proteases has not been previously conducted. This emphasizes the originality of the study. 

## 2. Study Design 

### 2.1. Selection of the Studies in the Literature

First, the text provides an overview of corn production and how its protein is obtained. It also discusses the concept of bioactive peptides and their relationship to non-communicable chronic diseases. Then, the literature was reviewed to find studies on corn bioactive peptides. Only articles and books published in English were evaluated. The search was performed using Scopus, Web of Science, Wiley, and Medline/PubMed using the terms “corn bioactive peptides” and “zein bioactive peptides”. Inclusion criteria comprised studies from the last 10 years (2014—September/2024). Therefore, 80 scientific research articles fit the inclusion criteria and were analyzed. The exclusion criteria included studies that did not provide relevant information on the health benefits of these peptides. A total of 70 scientific articles were excluded. The information was gathered from databases such as Scopus, Web of Science, Google Scholar, Science Direct, Lilacs, and SciELO.

The studies were categorized into three biological activities: antioxidant, hypertensive (ECA), and other activities (hepatic protection for alcohol, antimicrobial, anti-cancer, cell-penetrating capability), and presented in a graphical format.

### 2.2. Use of Database to Predict Bioactive Peptides

Amino acid sequences of α-zein-related corn storage proteins were acquired from the UniProtKB database [14]. The database was accessed in 2024. The protein name and its respective species were entered in UniProtKB: https://www.uniprot.org/uniprot/ (accessed on 8 August 2024).

A search was conducted using the terms “alpha zein *Zea mays*” and “alpha zein corn” to verify all amino acid sequences of α-zein entries in the database. Only protein amino acid sequences with a level of transcription were included based on the database’s classification system. In cases where multiple sequences shared the same protein name, the BLAST tool: https://www.uniprot.org/blast/ (accessed on 8 August 2024) was utilized to compare their identities. Sequences with an identity of 90% or higher were considered the same, while those with an identity of less than 90% were treated as different amino acid sequences.

The amino acid sequences representing α-zein (Table 1) were analyzed using BIOPEP-UWM: https://biochemia.uwm.edu.pl/biopep/start_biopep.php (accessed on 10 August 2024) to identify potential bioactive peptides in proteins. To conduct the in silico bioactivity analysis, “Protein”, “Profiles of potential biological activity”, and “For your sequence” were selected. The amino acid sequence obtained from the UniProtKB database was inserted, and then the “Report” option was selected. Peptide activity was tabulated.

The BIOPEP-UWM database was also used for two simulations of gastrointestinal digestion. For the first simulation, the enzymes pepsin (EC 3.4.23.1), trypsin (EC 3.4.21.4), and chymotrypsin (EC 3.4.21.1) were selected. The second simulation was performed only with subtilisin (EC 3.4.21.1). For the simulations, the following options were selected: “Protein”, “Analysis”, “Enzyme action”, and “For your sequence”. Sequences of amino acids obtained from UniProtKB were inserted, the enzymes were chosen, and the option “View the report with the results” was selected. Peptide fragments that were predicted to exhibit biological activity in the gastrointestinal simulation were tabulated. The frequency of occurrence of bioactive fragments (A) with some biological activity, such as antioxidant and ACE, DPP-IV, and DPP-III inhibition, was obtained from the database by clicking on “Calculations” and using the following equation: *A* = *a*/4*N*, where *a* is the number of fragments in the protein amino acid sequence with a given activity. *N* is the number of amino acid residues in the storage protein.

PeptideRanker: http://distilldeep.ucd.ie (accessed on 12 August 2024) was used to classify the probability of activity of peptides obtained from BIOPEP-UWM (Table 2 and Table 3) [17]. PeptideRanker classifies peptides according to the probability of being bioactive using a score from 0 to 1. The closer to 1, the more likely that the amino acid sequence has biological activity. The database was accessed in 2024. Any peptide predicted to have a score above 0.5 is labeled as bioactive by the database, but at a threshold of 0.8, false-positive rates for long and short peptides are reduced by 11% and 16%, respectively; at a threshold of 0.5, false-positive rates are reduced by 2% and 6%, respectively. Given this information, in the current study, only peptides with a score greater than 0.8 in PeptideRanker were considered and compared with peptides reported in the literature.

## 3. Corn Production, Processing and Composition

According to the Foreign Agricultural Service [18], world corn production reached 1.22 billion tons in 2024. The United States of America is the largest global producer of this cereal, with a projected harvest of 386.97 million tons [19]. Corn is the primary source of industrial starch and is also used as a raw material in the production of several food products, such as cornmeal, corn flour, corn flakes, hominy, and polenta; this staple crop forms the basis of human and non-human animal diets in several countries [20]. The importance of corn in the human diet is related to the nutritional properties of the grain: it contains 72.3% total carbohydrates (60% of which is starch) [21], 9.29% proteins, 3.75% lipids, 1.19% total minerals, and 1.99% fibers [22]. 

Dry or wet routes carry out industrial processing of corn. Dry processing is used to obtain products such as corn flour, snacks, and breakfast cereals, whereas wet processing can be used to produce starch, corn syrup, glucose, dextrin, and corn gluten meal [23]. Corn gluten meal is the dehydrated residue resulting from the separation of starch, which has a high protein content (about 68%). This protein fraction is mainly formed by zeins (~68%), the corn prolamins. The zeins are obtained solely from the grain endosperm. Zeins are a source of methionine and cysteine but have low amounts of lysine and tryptophan [24,25]. In addition, corn gluten meal contains about 28% glutelins and 1.2% albumins and globulins [8,26]. 

The proximate composition of corn may vary according to grain cultivar, crop system, and industrial processing. Zeins can be divided into α-, β-, γ- and δ-zeins, which differ in solubility and ability to form disulfide bonds [27]. The primary form found in corn is α-zein, corresponding to 80% of total zeins in the raw material. Corn α-zeins can be divided into two classes with different molecular masses, 22 and 24 kDa, known as Z19 and Z22, respectively. β-Zein, which represents 10% of the total zeins in corn, has a molecular mass of 15 kDa. γ-Zein can have a molecular mass of 16, 27, or 50 kDa, and δ-zein can have a molecular mass of 10 or 18 kDa [27]. Zein contains a high proportion of non-polar amino acids, such as glutamine, leucine, proline, and alanine [28], which results in a structural conformation with a highly hydrophobic surface. The amino acid sequence of zein is rich in sulfur-containing amino acids. Additionally, it has an unbalanced nitrogen content due to the deficiency in both acidic and basic amino acids. Because of the presence of many uncharged amino acids, zein is insoluble in water [29].

Commercially available zeins are poorly soluble in water but soluble in ethanol, acetone, and alkaline solutions (pH greater than 10), and their fractions have different solubilities in different solutions. α-Zein is soluble in the solution of ethanol, and β-zein is soluble in 60% ethanol and insoluble in 95% ethanol. Alcoholic solutions containing reducing agents are used for the solubilization of γ-zein. δ-Zein has a similar solubility to α-zein [30]. Kasaai [31] described that the low solubility of zein in water is related to its globular structure and the relevant presence of non-polar amino acids, particularly leucine, alanine, and proline, which make up 50% of the protein. Given its amino acid composition, zein is classified as a hydrophobic protein.

## 4. Obtention of Bioactive Peptides

Bioactive peptides are generated mainly from food proteins and have between 2 and 20 amino acids and a molecular mass of less than 6 kDa [32]. Zaky et al. [32] described that bioactive peptides exert a positive effect on several human body functions, such as reducing blood pressure, through peptides that inhibit the action of angiotensin-converting enzyme (ACE) (EC 3.4.15.1) and reducing blood glucose levels, through peptides that inhibit dipeptidyl peptidase IV (DPP-IV) (EC 3.4.14.5). According to Zaky et al. [32], bioactive peptides can also act as antioxidant, antibacterial, immunomodulatory, and antithrombotic agents.

The advantages of ingesting peptides, as compared to proteins, are related to their lower molecular mass, less complex structure, and increased digestibility by gastrointestinal enzymes [33]. As stated by Chinnadurai et al. [34], peptides are non-toxic and cause fewer side effects compared to synthetic inhibitors. Peptide bioactivity is dependent on the nature of the protein substrate, enzyme specificity, and hydrolysis conditions [35].

### 4.1. Production of Bioactive Peptides

Bioactive peptides can be produced through various methods, including chemical processes using acids and bases, enzymatic methods using proteolytic enzymes, and microbiological processes involving fermentation by proteolytic bacteria [36]. Enzymatic processes for hydrolyzing proteins into peptide amino acid sequences require specific conditions, such as controlled pH and temperature, which depend on the enzyme used. Enzymatic hydrolysis offers the advantage of not using chemicals, unlike chemical hydrolysis. Enzymatic methods can produce different peptide amino acid sequences with the ability to modulate various biological functions. In comparison, microbiological methods are less flexible and require longer processing times than enzymatic methods [36]. The main enzyme used to produce α-zein hydrolysates and obtain the bioactive peptides is alcalase [37,38,39,40], although other enzymes can also be used, such as thermolysin [41]. 

Ulug et al. [42] noted that new methods are being developed to produce bioactive peptides, such as high hydrostatic pressure processing, ultrasound, microwave-assisted extraction, and subcritical water hydrolysis. The high hydrostatic pressure technique is particularly interesting because, by combining high pressures with low temperatures, it is possible to decrease the impact on protein and peptide conformation [42].

Tian et al. [43] stated that ultrasound can increase the conversion of proteins to peptides and decrease the time required for hydrolysis because of the influence of mechanical waves on protein structure, mainly related to the breaking of non-covalent bonds. Peptide extraction by microwave techniques involves the transfer of energy from microwaves to molecular interactions by mechanisms of dipole rotation and ionic conduction [42]. 

According to Ulug et al. [42], the use of subcritical water at temperatures of 100 to 374 °C and pressures lower than 22 MPa (the critical point of water) is an interesting method for extraction of peptides from food proteins because it does not generate waste. Zhang et al. [44] used this method on corn zein and demonstrated that peptides were efficiently released.

### 4.2. Enzymatic Hydrolysis of Proteins

The production of bioactive peptides by enzymatic hydrolysis occurs in two steps. In the first step, enzymes are in their native state and bind to the protein complex. Then, from the enzyme-protein interaction, enzymatic hydrolysis begins. In the second step, the enzyme cleaves the protein at specific points, according to enzyme specificity. It releases peptide fragments with different molecular masses as well as some free amino acids [45].

The degree of protein hydrolysis and the characteristics of the resulting peptides depend on enzyme specificity. According to Wang et al. [46], the proteases most used in in vitro studies for the simulation of gastrointestinal digestion are pepsin, pancreatin (which is a mixture of several digestive enzymes, including trypsin and chymotrypsin), and chymotrypsin. Trypsin is responsible for cleaving proteins and peptides from the carbon side of arginine and lysine residues. Chymotrypsin cleaves proteins and peptides from the carboxyl side of aromatic and hydrophobic amino acids, such as phenylalanine, tyrosine, and tryptophan. Pepsin, present in the stomach mucosa, cleaves bonds of peptides that contain phenylalanine, leucine, or glutamic acid at the carboxyl end [47]. Another enzyme often used to hydrolyze proteins in order to obtain bioactive peptides is the subtilisin, which is an endo protease from the group of serine proteases and is secreted by *Bacillus amyloliquefaciens*. Peterle et al. [48] described that the enzyme subtilisin cleaves peptide bonds between large uncharged amino acid residues.

## 5. Non-Communicable Chronic Diseases

Chronic hyperglycemia, diagnosed by a fasting glucose level above 100 mg/dL, can result in a metabolic disease known as type 2 diabetes mellitus, which is characterized by insulin resistance. In this case, muscle, fat, and liver cells do not respond adequately to insulin, resulting in difficulty absorbing glucose into the blood [49,50]. According to the International Diabetes Federation [12], about 483 million people worldwide have type-2 diabetes mellitus. Bioactive compounds present in drugs marketed for the control of diabetes, such as sitagliptin and alogliptin, act by inhibiting the enzyme DPP-IV or mimicking incretins, which are hormones responsible for stimulating insulin secretion by pancreatic β cells in the presence of glucose. The most important incretins are glucagon-like peptide (GLP-1) and glucose-dependent insulinotropic peptide (GIP) [51]. Peptides with DPP-IV inhibition properties can maintain incretin activity and promote insulin secretion, contributing to the management of diabetes [50].

The enzyme DPP-III plays crucial roles in the endogenous system related to pain modulation and reduction of oxidative stress. Furthermore, DPP-III was shown to contribute to the activation of transcription factor Nrf2, one of the important factors in regulating the expression of molecules that have an anti-inflammatory and antioxidant role, such as NAD(P)H and NQO1 [52].

High blood pressure, also known as hypertension, is defined as sustained elevation of systolic/diastolic blood pressure to levels above 140 and/or 90 mmHg. This condition must be managed closely to reduce the risk of cardiovascular diseases. Physiological control of blood pressure depends on ACE, which participates in the regulation of the renin-angiotensin-aldosterone system and the kallikrein-kinin system. The ACE enzyme is responsible for converting angiotensin I to angiotensin II, which promotes the release of aldosterone and reactive oxygen species, causing an increase in blood pressure. Consequently, ACE inhibitors, α and β receptor blockers, and angiotensin II receptor antagonists contribute to maintaining normal blood pressure and decreasing the risk for hypertension and cardiovascular disease [12,53].

Reactive oxygen species (ROS), such as superoxide anions, hydrogen peroxide, hydroxyl radicals, and singlet oxygen, are naturally produced by the body under homeostasis. However, in excess, ROS can lead to the emergence of numerous degenerative diseases (e.g., cardiovascular diseases, chronic obstructive pulmonary disease, chronic kidney disease, neurodegenerative diseases, cancer, and diabetes) because of their strong interaction with essential regulatory components of cells [54,55]. Consumption of a diet rich in antioxidant compounds is recommended to minimize the deleterious effects of ROS [56].

## 6. Studies Reported in the Literature

**Figure 1:**
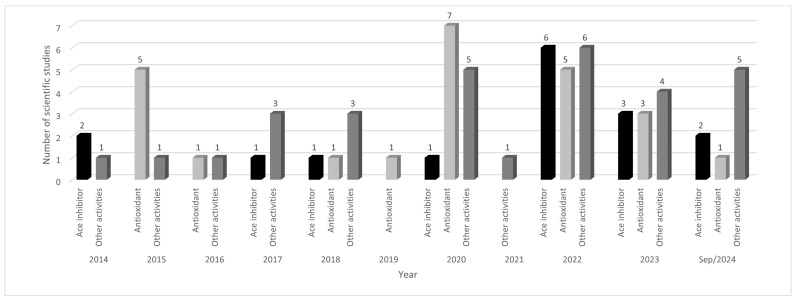
Number of scientific studies on bioactive peptides in corn in the last ten years, according to biological activity. Legend: Other activities include hepatoprotection (facilitating alcohol), antimicrobial, anti-cancer, and cell-penetrating capability.

Over the past 10 years, there has been an increase in scientific studies related to the biological activity of corn peptides, indicating a possible increase in the characterization of these molecules (Figure 1). Between 2014 and 2018, an average of 4.0 articles was published per year. However, in the five years from 2019 to September 2024, this average increased to approximately 10.0 articles per year. This significant increase was attributed to the potential biological effects of α-zein peptides, which contributed to a higher number of articles published in the last five years compared to the previous five years.

All studies were performed on corn gluten or zein isolated from corn gluten. Currently, there are no studies identifying zeins according to their classification as α, β, γ, or δ. Most studies performed enzymatic hydrolysis, as did Jing et al. [7], for the identification of peptides with antioxidant activity in Caco-2 cells in corn gluten meal. One study performed chemical hydrolysis using the Fmoc method, identifying two peptides with antioxidant activity in Caco-2 cells [57], and another performed microbiological hydrolysis (*Bacillus subtilis* MTCC5480), detecting peptides with antioxidative action [58].

Corn peptides were mainly studied for their antioxidative potential. Other biological properties, such as liver protection, were reported. Corn peptides hydrolyzed with alcalase reduced the levels of alanine transaminase/aspartate transaminase, laminin, and collagen type IV and III in serum and increased albumin and antioxidant capacity in the liver of rats [59]. Salouti et al. [60] found that corn peptides acted against *Staphylococcus aureus* proliferation in wounds when applied with silver nanoparticles. Ortiz-Matinez et al. [8] studied corn peptides hydrolyzed with alcalase and identified anti-cancer activity against HepG2 cells. Furthermore, the authors found that apoptosis induction rates increased 4-fold in cancer cells.

### 6.1. In Vitro Evidence of Health Benefits Associated with Corn Bioactive Peptides

Díaz-Gómez et al. [16] selected three peptide amino acid sequences based on the analysis of data available in BIOPEP-UWM. The sequences were named as follows:

(1) 19ZP1 (FNQLAALNSAAYLQQQQLLPFSQLA)
(2) 19ZP2 (QLADVSPAAFLTQQQLLPFYLHAM)
(3) 19ZP3 (AYLQAQQLLPFNQLVRSPAA)

The authors conducted an in silico study and found that the probability of the selected peptides exhibiting anti-cancer activities was below 0.01 because of limitations in cell penetration. Accordingly, no anti-cancer activity was detected in in vitro studies. On the other hand, antioxidant and ACE inhibitory activities were identified in both in silico and in vitro studies. Sharma et al. [61] described an ACE inhibitory action of 98.76 ± 1.28% on alcalase hydrolyzed corn peptides, with the amino acid sequences represented by DPANLPWG, FDFFDNIN, WNGPPGVF, and TPPFHLPPP.

In a study by Chanajon et al. [62], the ACE inhibitory potential of corn gluten peptides produced with porcine pepsin enzyme was analyzed. They observed a decrease of 59 mmHg in rats treated with a corn peptide diet compared to the control group (diet without peptide increment) after six weeks. Wu et al. [63] described that corn peptides contributed to preventing myocardial damage in rats during excessive swimming exercise. 

Qu et al. [5] developed a corn peptide chelated with iron through the action of neutrose and alcalase enzymes. Subsequently, they applied ultrasound and reacted it with iron chloride, resulting in 82.21% ACE inhibitory activity. 

The antioxidant properties of peptides are related to hydrophobic amino acids, such as tyrosine, phenylalanine, leucine, and alanine, an effect stemming from the ability of these compounds to donate hydrogen [4]. Wang et al. [64] analyzed corn peptides hydrolyzed using the commercial enzymes Alcalase and Protamex. The authors found that alcalase can release peptides with greater antioxidant capacity through the DPPH•, ABTS+, and hydroxyl radical techniques compared with peptides produced by Protamex. Another study found that a combination of alcalase and trypsin immobilized on a calcium–quinine–alginate transporter was able to hydrolyze corn proteins into highly stable peptides [64].

Wang et al. [6] discovered four corn gluten peptides (WIY, YLW, LAYW, and LYFY) with antioxidant capacities. These peptides exhibited ABTS activity 2.58–3.26 times higher and ORAC activity 5.19–8.63 times higher than Trolox. According to Wang et al. [65], the sequence YFCLT present in corn gluten meal exhibits excellent DPPH• scavenging activity. Some peptide amino acid sequences from corn gluten meal, such as FPLEMMPF, QQPQPW, and CSQAPLA, have also been reported to be excellent antioxidants [64,66,67]. Hu et al. [68] studied the hydrolysis of corn gluten proteins in vitro using three distinct enzymes, namely papain, ficin, and bromelin, and observed that the resulting peptides showed antioxidant potential. However, antioxidant action was dependent on the enzyme and hydrolysis conditions applied.

### 6.2. In Vivo Evidence of Health Benefits Associated with Corn Bioactive Peptides

Li et al. [69] described that the peptides hydrolyzed from corn silk (stigma) extract showed ACE inhibitory activity in vivo, reducing the blood pressure of rats by 36.78 ± 13.25 mmHg after 1 h of injection. Guo et al. [70] analyzed corn germ meal peptides hydrolyzed in an enzyme membrane reactor and observed that rats treated with the peptides had a decrease in blood pressure of 18.22 mmHg compared with the control group.

According to Liu et al. [30], the bioactive peptides resulting from the degradation of zein present an angiogenic response in mice, contributing to tissue regeneration when applied to an injector biomaterial contributing to confirming the biological activity of these compounds. Wang et al. [71] reported that zein peptides exhibited liver enzyme activities, including alcohol dehydrogenase, acetaldehyde dehydrogenase, and endogenous antioxidant enzymes, in addition to improving glutathione and triacylglycerol levels.

Hira et al. [10] reported that papain-hydrolyzed zein induced strong secretion of GLP-1 in the ileum of anesthetized rats. Peptide administration decreased DPP-IV activity by 26.8%, resulting in a reduction in plasma glucose levels through a 3.1-fold increase in GLP-1 release and a 6.3-fold increase in insulin secretion compared with the control.

### 6.3. In Silico Approach to Investigate Bioactive Peptides

Before carrying out in vitro and in vivo studies, it is possible to analyze peptide amino acid sequences and their biofunctions by using an in silico approach. The advantages of in silico studies include economy of time and reagents and the possibility of determining which peptides have biological potential. Databases contain the following information on peptides: sequence expressed in one-letter code, according to the amino acid name, name, length of the peptide chain expressed in the number of amino acid residues, source of origin, molecular mass, and reference article [72]. Databases also allow searching for exact correspondence of an amino acid sequence of interest [72].

BIOPEP-UWM is an important tool for investigating proteins as potential sources of bioactive peptides. BIOPEP-UWM comprises two peptide databases, one with over 3700 peptides exhibiting various biological functions and a second with 480 sensory peptides, including amino acids with aromatic characteristics [73]. According to Iwaniak et al. [73], sensory peptides are substances that have an experimentally verifiable flavor and, therefore, can influence food taste.

Another database of active peptide amino acid sequences is UniProt, which provides access to proteomes of more than 247,000 species with full genome representation [14]. The bank extracts information from scientific publications and stores it in the UniProtKB/Swiss-Prot section of the UniProt Knowledgebase. The data describe functional information in the form of controllable syntax summaries and readable free text, such as Gene Ontology (GO) and ChEBI [14]. UniProt is simpler than BIOPEP-UWM, but the combined use of both databases can provide interesting results, given that an amino acid sequence of bioactive peptides may be reported in one database but not in the other.

As underscored by Minkiewicz et al. [17], after obtaining amino acid sequences from the BIOPEP-UWM database, it is possible to evaluate the probability of their biological action by using the PeptideRanker database. Probability is estimated considering the charge distribution of the peptide and amino acid sequence. The determination of peptide bioactivity is based on enzyme specificity and the recognition of the amino acid sequence [17]. These mathematical tools, therefore, help to determine what can be expected in in vitro and in vivo assays. Iwaniak et al. [72] described that the in silico approach may also be used after in vitro protein hydrolysis to assist in the identification of hydrolyzed peptides and predict their bioactivity.

### 6.4. In Silico Prediction of Bioactive Peptides from Corn Storage Proteins

**Figure 2:**
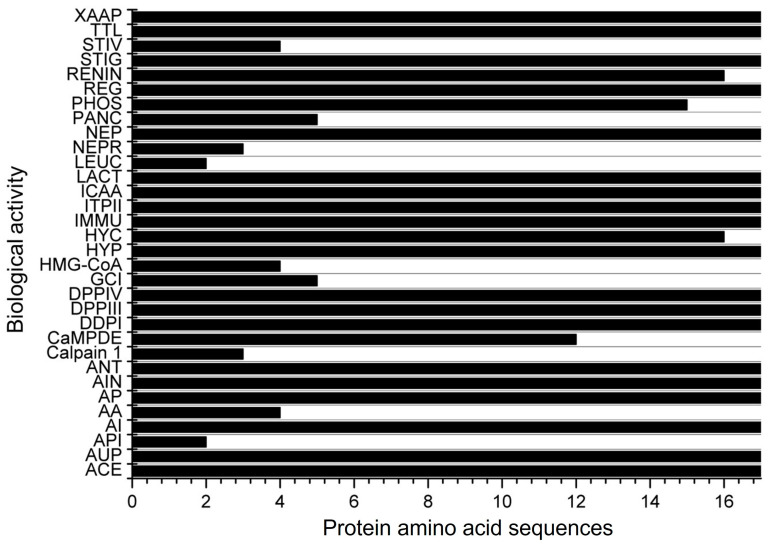
Biological activities of the 17 α-zein protein amino acid sequences analyzed using the BIOPEP-UWM database. Legend. ACE: ACE inhibitor, AUP: activating ubiquitin-mediated proteolysis, API: acylaminoacyl peptidase inhibitor, AI: alpha-glucosidase inhibitor, AA: antiammnestic, AP: antibacterial peptide, AIN: anti-inflammatory, ANT: antioxidative, Calpain 1: calpain 1 inhibitor, CaMPDE: CaMPDE inhibitor, DDPI: D-Ala-D-Ala dipeptidase inhibitor, DPPIII: dipeptidyl peptidase III inhibitor, DPPIV: dipeptidyl peptidase IV inhibitor, GCI: glutamate carboxypeptidase inhibitor, HMG-CoA: HMG-CoA reductase inhibitor, HYP: hypotensive, HYC: hypouricemic, IMMU: immunomodulating, ITPII: inhibitor of tripeptidyl peptidase II, ICAA: inhibitor of cytosol alanyl aminopeptidase, LACT: lactocepin inhibitor, LEUC: leucyltransferase inhibitor, NEPR: neprilysin 2 inhibitor, NEP: neuropeptide, PANC: pancreatic lipase inhibitor, PHOS: phospholipase A2 inhibitor, REG: regulating (phosphoglycerate kinase activity), RENIN: renin inhibitor, STIG: stimulating (glucose uptake stimulating peptide), STIV: stimulating (vasoactive substance release), TTL: tubulin-tyrosine inhibitor, XAAP: Xaa-Pro inhibitor.

A total of 17 amino acid sequences for α-zein from Z. mays were identified under the transcriptional evidence level at UniProtKB (Table 1). Peptides derived from these proteins were assessed for potential biological activity in the BIOPEP-UWM database (Figure 2). It was found that these peptides have 32 possible biological actions, including DPP-IV and DPP-III inhibition, ACE inhibition, vasodilator action, activation of ubiquitin, and inhibition of HMG-CoA reductase, among others. However, no hepatoprotective or antimicrobial activity was found in the database despite being reported in some studies in the literature over the past decade. From the 32 possible biological activities found, 19 were observed in all 17 protein amino acid sequences (Figure 2).

**Figure 3:**
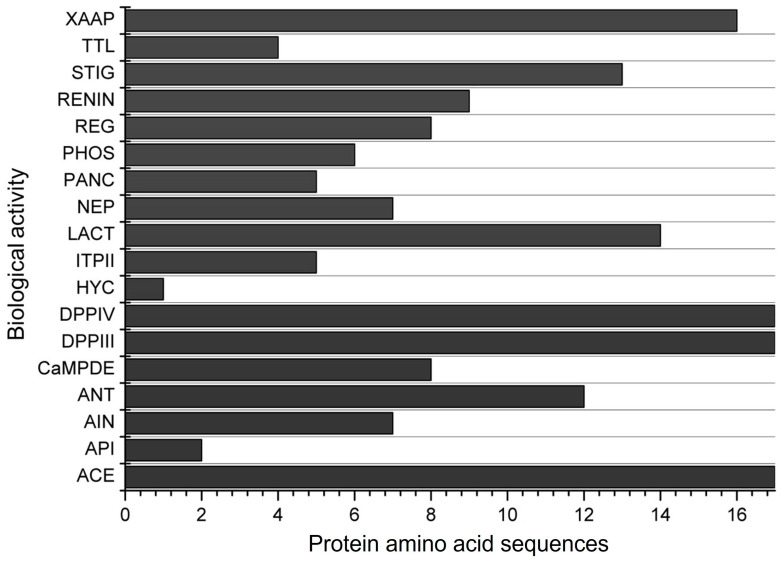
Biological activities of the 17 α-zein protein amino acid sequences analyzed using the BIOPEP-UWM database after simulated gastrointestinal digestion with pepsin, trypsin, and chymotrypsin. Legend. ACE: ACE inhibitor, API: acylaminoacyl peptidase inhibitor, AIN: anti-inflammatory, ANT: antioxidative, CaMPDE: CaMPDE inhibitor, DPPIII: dipeptidyl peptidase III inhibitor, DPPIV: dipeptidyl peptidase IV inhibitor, HYC: hypouricemic, ITPII: inhibitor of tripeptidyl peptidase II, LACT: lactocepin inhibitor, NEP: neuropeptide, PANC: pancreatic lipase inhibitor, PHOS: phospholipase A2 inhibitor, REG: regulating (phosphoglycerate kinase activity), RENIN: renin inhibitor, STIG: stimulating (glucose uptake stimulating peptide), TTL: tubulin-tyrosine inhibitor, XAAP: Xaa-Pro inhibitor.

The in silico simulation of gastrointestinal digestion of the 17 α-zein proteins using pepsin, trypsin, and chymotrypsin, performed in the BIOPEP-UWM, resulted in peptide sequences exhibiting 18 different biological actions (Figure 3). All α-zein protein sequences analyzed in this simulation showed activities related to ACE, DPP-IV, and DPP-III inhibition, as observed in the previous evaluation (Figure 2). Peptides with antioxidant activity were observed in 12 of the 17 proteins analyzed. Only 1 protein sequence among the 17 analyzed showed hypouricemic action.

**Figure 4:**
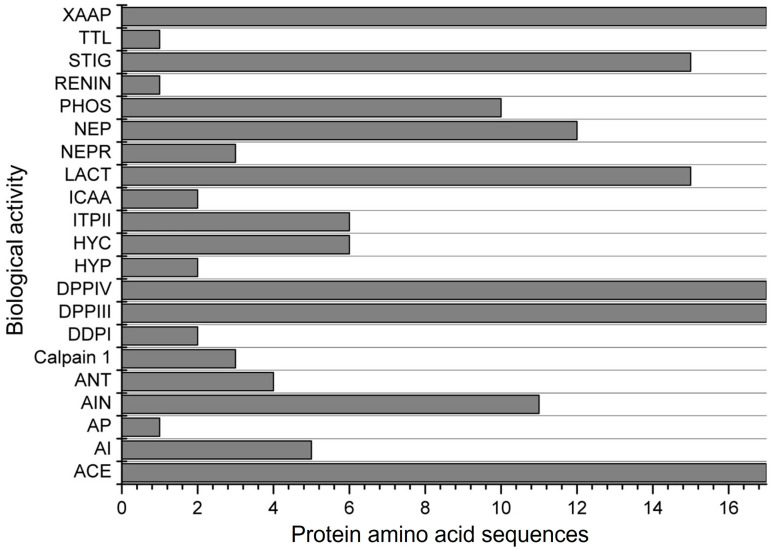
Biological activities found for the 17 α-zein protein amino acid sequences analyzed by using the BIOPEP-UWM database after simulated digestion with subtilisin. Legend. ACE: ACE inhibitor, AI: alpha-glucosidase inhibitor, AP: antibacterial peptide, AIN: anti-inflammatory, ANT: antioxidative, Calpain 1: calpain 1 inhibitor, DDPI: D-Ala-D-Ala dipeptidase inhibitor, DPPIII: dipeptidyl peptidase III inhibitor, DPPIV: dipeptidyl peptidase IV inhibitor, HYP: hypotensive, HYC: hypouricemic, ITPII: inhibitor of tripeptidyl peptidase II, ICAA: inhibitor of cytosol alanyl aminopeptidase, LACT: lactocepin inhibitor, NEPR: neprilysin 2 inhibitor, NEP: neuropeptide, PHOS: phospholipase A2 inhibitor, REG: regulating (phosphoglycerate kinase activity), RENIN: renin inhibitor, STIG: stimulating (glucose uptake stimulating peptide), TTL: tubulin-tyrosine inhibitor, XAAP: Xaa-Pro inhibitor.

Another evaluation involved simulating the subtilisin digestion of α-zein proteins. Subtilisin is an endopeptidase commercially known as alcalase, which is highly used in in vitro assays to produce bioactive peptides. The simulation resulted in peptides with 21 possible biological actions (Figure 4). Only 4 activities were found for all 17 protein sequences evaluated. The peptides obtained were found to inhibit ACE, stimulate glucose uptake, and inhibit DPP-IV, DPP-III, and Xaa-Pro. Among the 17 proteins analyzed, peptides with antioxidant activity were observed in only 4, a lower proportion compared to simulated digestion with pepsin, trypsin, and chymotrypsin. However, the simulation with subtilisin revealed peptides with alpha-glucosidase inhibitor, antibacterial peptide, calpain 1 inhibitor, D-Ala-D-Ala dipeptidase inhibitor, hypotensive action, inhibitor of cytosol alanyl aminopeptidase, and neprilysin 2 inhibitor. These results were not observed in the gastrointestinal digestion simulation.

Among those 17 α-zein protein amino acid sequences obtained from UniProt, two proteins, coded as P06678 and P04705 did not show more than 90% similarity with any other protein, indicating that their amino acid sequences were different from all others. Additionally, proteins Q94IM1 and P04698 had a similarity greater than 90%, and the same was true for proteins P0679 and P04700. Therefore, only the proteins Q94IM1 and P06679 were considered to be different from the others. The remaining proteins selected were P06674 and P06676 because they showed a similarity higher than 90% with the other proteins, although they were not similar to each other. The amino acid sequences of these selected proteins are described in Table 2 and Table 3. They were grouped into 19-kDa α-zein (P06674, P06676, P06678, and P04705) and 22-kDa α-zein (P06679 and Q94IM1). Both tables also display the bioactive peptide amino acid sequences obtained from evaluating the potential biological activities of the proteins and simulating gastrointestinal and subtilisin digestions in silico by the BIOPEP-UWM database. According to Garcia, Barros, and Rocha (2020), for potential biological activities, BIOPEP-UWM is able to predict protein fragments with biological activity. Still, it does not simulate the hydrolysis of protein chains using a specific mechanism. Therefore, it identified numerous potential biological activities but with a low frequency of occurrence, as shown in Table 2 and Table 3. For example, the probability of a peptide exhibiting CaMPDE inhibitory action was 0.0043, found for the protein P06674 (Table 2). High frequencies of occurrence were only observed for biological functions resulting from numerous amino acid sequences, such as DPP-IV inhibition, with a frequency of occurrence of 0.8130.

As described by Wang et al. [46], enzymes cleave proteins at different points according to their specificity; thus, different peptides are obtained by digestion with different enzymes. Therefore, simulated gastrointestinal digestion afforded fewer fragments with different biological activities than the evaluation of the potential biological profile of non-hydrolyzed proteins (Table 2 and Table 3). However, it is essential to consider the potential that proteins can provide in terms of biological activity for health benefits. There are alternative methods, aside from enzymatic hydrolysis, to obtain bioactive peptides, such as physical processing.

The α-zein protein is made up of homologous α-helix structures, but its secondary structure depends on the solvent used to dilute the protein [74]. Several models have been proposed, such as ribbon [75], hook, and superhelix formats [76]. The model proposed by Argos et al. [75] is still accepted today and indicates that α-zein contains amino acid sequences that are highly hydrophobic, which can aggregate and form fibers or associate with membranes, remaining in a helical configuration. The authors proposed that there is a cluster of α-helices inside a distorted cylindrical surface covered by polar glutamine residues, which allow for hydrogen bonding, hydrophobic van der Waals interactions, and intermolecular packing, thereby maintaining the protein’s compact structure. The compact format of α-zein confirms that each enzyme can hydrolyze only a part of its structure, explaining why the proportion of bioactive peptides was higher when analyzing the potential bioactivity profile of non-digested proteins than when assessing enzymatically digested proteins. 

Bioactive di- and tri-peptides related to α-zein showed antioxidant and ACE inhibitory capabilities. ACE inhibitor peptides act by decreasing angiotensin II production and, consequently, decreasing vasodilation and blood pressure [69]. Furthermore, peptides were found to be associated with inhibition of DPP-IV, a serine protease responsible for stimulating insulin secretion [77], and DPP-III, which is responsible for inhibiting chelating agents and thiol [78]. According to Khaket et al. [78], dipeptides formed by aromatic pairs with large aliphatic or basic amino acids have the highest DPP-III inhibitory potential. In addition to antioxidant activity, ACE, DPP-IV, and DPP-III inhibition activities were rarely explored in in vitro or in vitro assays in the studies reported in the literature over the past decade. Based on these findings, it can be inferred that conducting an in silico study before in vitro or in vivo evaluations could be advantageous. This preliminary study may help direct the search for and exploration of specific biological activities during material evaluation. Ultimately, this approach could be cost-effective and time-saving by aiding in determining the most effective direction for research efforts. 

Table 4 describes peptides with scores above 0.8 according to PeptideRanker. Peptides were obtained from the BIOPEP-UWM database considering the proteins of Table 2 and Table 3 before and after simulated digestion with a combination of enzymes (pepsin, trypsin, and chymotrypsin) and subtilisin. The peptide amino acid sequences represented in Table 4 were identified in literature studies, mainly for DPP-III, DPP-IV, and ACE inhibitor activities and antioxidants. Some peptide amino acid sequences with ACE, DPP-III, and DDP-IV inhibitory activity were not described in studies on corn proteins.

Fragments reported to promote ACE inhibition were composed of more than two or three peptides, and, in some cases, as for the tripeptide LPF, the isolated fragment had more than 20 amino acids (Table 4). Such a discrepancy can be attributed to differences in digestion conditions (hydrolysis time, temperature, and proteolytic enzymes). A peptide amino acid sequence with ACE inhibitory action and high occurrence (according to PeptideRanker) identified in a study on corn proteins was obtained by chemical isolation methods (Fmoc and Noc) [69]. There were no reports on the identification of this sequence in corn by enzymatic digestion. Yano et al. [79] identified the second amino acid sequence (LF) with the highest frequency of ACE inhibition. They were able to isolate it as a dipeptide via digestion with thermolysin at 37 °C for 3 h. The IF dipeptide, with a possibility of occurrence of 0.9491, was identified by hydrolysis of corn with alcalase at 55 °C for 4 h [67]. 

Kaur et al. [86] described that peptide structure, length, composition, and amino acid chain sequence are decisive for ACE inhibitory activity, as large peptides (>12 amino acids) may not bind to the active sites of the enzyme. Peptides with highly acidic amino acids, such as asparagine and glutamine, have a negatively charged network that is responsible for chelating zinc atoms and inactivating enzymes via chelation [87].

ACE inhibition results from the interaction of the enzyme with three hydrophobic amino acids (proline, histidine, and phenylalanine) at the C-terminus of the protein. Furthermore, aromatic or alkaline amino acids such as arginine, glycine, valine, alanine, and isoleucine present at the N-terminus of inhibitory peptides may contribute to ACE inhibition [88]. Zhu et al. [89] described that zein has a hydrophobic segment with a high proportion of non-polar amino acids, such as leucine (19.3%), proline (9.0%), and alanine (8.3%), explaining the high proportion of peptides with possible ACE inhibitory action and the numerous studies reporting the efficacy of these isolated peptides.

Peptides with antioxidative potential were enzymatically hydrolyzed by alkaline proteases, such as alcalase and Flavourzyme, and were found to contain few amino acids in the sequences (3 to 7) (Table 4). Díaz-Gómez et al. [40] and Zhu et al. [89] reported that the antioxidant capacity of corn peptides is related to the presence of amino acids such as lysine, histidine, tyrosine, phenylalanine, proline, leucine, cysteine, and tryptophan. Peptides that have amino acids with hydrophobic and aromatic side chains act as hydrogen donors, contributing to the interruption of the peroxidation chain reaction [89].

Peptides with a frequency greater than 0.8 in PeptideRanker and potential DPP-IV and DPP-III inhibitory capacities were not identified in many studies, except LPP, VPL, YGGFM, and GGFL sequences. Mochida et al. [84] described that the DPP-IV inhibitory action of zein peptides might be related to the presence of proline and alanine at the N-terminus. However, according to Trinidad-Calderón et al. [3], the plausible mechanisms of these effects have not yet been studied in vitro. This is mainly due to the short in vivo half-life (minutes) of incretins and the fact that a continuous presence of inhibitors in the blood is required for analysis [90].

Based on the current research results, it is evident that bioinformatics is a cost-effective and efficient tool for designing, synthesizing, and selecting bioactive peptides. This is particularly beneficial for studying peptides with ACE inhibitory and antioxidative activities, as indicated by high scores from PeptideRanker. In addition, in silico evaluation can help in predicting peptides with DPP-IV inhibitory action from zein sources.

## 7. Conclusions

The present study revealed that prolamins present in corn, mainly the α-zein fraction, are good sources of proteins for the release of potentially bioactive peptides, especially those with ACE, DPP-IV, and DPP-III inhibitory activity and antioxidant action. Therefore, corn peptides can be considered a viable strategy to reduce the risk of type 2 diabetes mellitus, hypertension, and other diseases related to oxidative stress, such as cancer and inflammation. Additionally, it’s important to consider the by-product of corn starch production, known as corn gluten meal. This product is primarily composed of α-zein and is mainly used in animal feed. However, it represents a protein-rich raw material that can be utilized to obtain bioactive peptides suitable for human consumption. This study contemplates the utilization of the in silico approach to better usage of this valuable by-product. The current study also highlights the potential of using computational prediction as a powerful and cost-effective tool for designing and synthesizing new bioactive peptides from food sources. By utilizing advanced computational techniques, researchers can efficiently identify and predict peptides with potential biological activities, thus streamlining the development process. In particular, biocomputational tools are crucial for exploring bioactive peptides from corn zein proteins. These tools enable researchers to model and predict which peptides derived from corn zein may exhibit beneficial biological effects. Once identified, these peptides can be synthesized, or the proteins can be subjected to enzymatic hydrolysis to obtain smaller, bioactive fragments. The integration of computational predictions with enzymatic hydrolysis not only accelerates the discovery of new peptides but also ensures that the peptides are optimized for various applications, including nutraceuticals. This approach can lead to the development of innovative dietary supplements or functional foods with targeted health benefits, showcasing the significant impact of computational tools on the advancement of food science and nutrition.

## Figures and Tables

**Table 1:** Percentage of the identity of the alpha-zein protein amino acid sequences obtained from the UniProtKB database using the BLAST tool.

*Zea mays*—Alpha Zein																				
UniProt Data			Identity (BLAST)																	
Protein Name	UniProt Code	Length		Q94IM1	P04698	P06679	P04700	P06678	P04704	P06676	P04703	P06674	P06675	P04702	P02859	P04705	Q548E6	Q548E7	B6SIF7	B6SHV3
22 kDa alpha-zein 14	Q94IM1	266	Q94IM1	-	**98.9%**	88.9%	86.2%	49.1%	44.2%	46.7%	46.7%	43.6%	46.0%	46.7%	46.4%	41.9%	46.4%	45.5%	43.8%	44.0%
22 kDa alpha-zein 14	P04698	267	P04698		-	89.3%	87.3%	49.1%	44.4%	46.7%	46.7%	43.8%	43.5%	46.7%	43.9%	41.7%	46.4%	45.3%	43.7%	43.9%
22 kDa alpha-zein 8b	P06679	261	P06679			-	**90.1%**	49.1%	43.5%	42.4%	46.0%	42.9%	42.8%	46.8%	43.1%	43.2%	40.6%	45.4%	43.1%	43.1%
22 kDa alpha-zein 16	P04700	263	P04700				-	48.9%	44.0%	46.7%	46.7%	40.7%	42.8%	47.1%	43.5%	42.3%	46.7%	43.1%	42.5%	42.8%
Zein-alpha 19D1	P06678	240	P06678					-	56.0%	58.5%	58.5%	56.3%	56.0%	58.1%	56.5%	48.7%	58.1%	52.2%	56.5%	56.5%
Zein-alpha ZG99	P04704	235	P04704						-	71.8%	71.8%	**91.4%**	**91.1%**	69.8%	**92.3%**	87.2%	71.0%	87.3%	**91.9%**	**91.5%**
Zein-alpha 19C1	P06676	240	P06676							-	**99.6%**	70.5%	72.5%	**96.7%**	72.1%	61.4%	**99.9%**	65.4%	72.1%	72.1%
Zein-alpha A20	P04703	240	P04703								-	70.5%	72.5%	**96.3%**	72.1%	61.4%	**98.3%**	62.8%	72.1%	72.1%
Zein-alpha 19A2	P06674	230	P06674									-	**90.5%**	68.5%	**92.2%**	79.3%	69.7%	68.7%	**91.8%**	**90.9%**
Zein-alpha 19B1	P06675	234	P06675										-	70.5%	**97.4%**	81.8%	71.7%	80.1%	**96.6%**	**99.6%**
Zein-alpha M6	P04702	240	P04702											-	70.1%	57.9%	**97.9%**	63.9%	70.1%	70.1%
Zein-alpha A30	P02859	234	P02859												-	82.4%	71.3%	80.5%	**98.3%**	**97.9%**
Zein-alpha PZ19.1	P04705	186	P04705													-	60.4%	87.8%	81.3%	82.4%
19kD alpha zein B3	Q548E6	240	Q548E6														-	**97.0%**	71.3%	80.5%
19kD alpha zein B2	Q548E7	267	Q548E7															-	80.1%	**97.0%**
Zein-alpha PMS1	B6SIF7	234	B6SIF7																-	**97.0%**
Zein-alpha 19B1	B6SHV3	234	B6SHV3																	-

Sequences with an identity of 90% or higher were considered the same.

**Table 2:** Predicted potential for obtaining bioactive peptides for α-Zein with 19 kDa.

α-Zein—19 kDa						
**P06674**—Protein amino acid sequence: KIFCFLMLLGLSASAATATIFPQCSQAPITSLLPPYLSPAVSSVCENPILQPYRIQQAIAAGILPLSPLFLQQ PSALLQQLPLVHLLAQNIRAQQLQQLVLGNLAAYSQQHQFLPFNQLAALNSAAYLQQQLPFSQLAAAYPQQFLPFNQLAALNSAAYLQQQQLPPFSQLADVSPAAFLTQQQLLPFYLHAAPNAGTVLQLQQLLPFDQLALTNPTAFYQQPIIGGALF						
**Activity**	**Profiles of potential biological activity**		**GI: pepsin, trypsin, chymotrypsin**		**Subtilisin**	
	**Bioactive fragment**	**A**	**Bioactive fragment**	**A**	**Bioactive fragment**	**A**
ACE inhibitor	PF, LPP, ILP, IR, AAP, LPP, LVL, FP, IRAQQ, VSP, LAA, LSP, LQP, IRA, YL, LF, FY, FNQ, AY, LSPA, YP, FYQQ, LLP, LQQ, HLL, PL, IA, AF, AP, LA, RA, AA, IF, IG, GI, GA, GL, AG, HL, GT, GG, AI, LG, TNP, FCF, CF, LQ, LN, PT, TQ, PP, PQ, AV, LPF, AFL, IL, QP, LPL, LQL, LP, GIL	0.5609	IR, PL, IF, GL, CF, PF	0.0261	PL, GL, CF, PF	0.0217
Antibacterial peptide	AA	0.0478				
Immunomodulating	PFNQL, FLPFNQL	0.0174				
Stimulating (glucose uptake stimulating peptide)	VL, LV, IL, II, LL	0.0565	VL	0.0043	VL	0.0087
Neuropeptide	YL, YR, PY, IL	0.0391				
Regulating (phosphoglycerate kinase activity)	SL	0.0043				
Anti-inflammatory	PY, LLPF, LPF	0.0391				
Hypotensive	AA, FY	0.0565				
Antioxidative	LH, HL, AY, LHA, IR, LLPF, FC, LPL, LQL, YSQ, YPQ, FY, YL, LT	0.1043	IR	0.0043		
Activating ubiquitin-mediated proteolysis	RA, LA	0.0348				
Alpha-glucosidase inhibitor	FY, YP, PP, AD, FY	0.0261			AD	0.0043
Dipeptidyl peptidase IV inhibitor	PP, LA, AP, PA, LP, LL, HA, SP, FP, YP, GA, IA, RA, NP, TA, QP, FL, HL, AL, SL, GL, LPL, AA, PL, LQP, AD, AF, AG, AS, AT, AV, AY, DQ, FN, GG, GI, II, IL, IQ, IR, KI, LH, LM, LN, LT, LV, ML, NA, NL, NQ, PF, PI, PN, PQ, PS, PT, PY, QA, QF, QH, QL, QN, QQ, RI, SV, TI, TN, TQ, TS, TV, VH, VL, VS, YL, YQ, YR, YS, APIT, LPF	0.8130	AL, GL, PL, IR, PF, QF, QL, TN, VH, VL	0.0739	PA, AL, GL, PL, AD, AS, ML, PF, QL, VL, VS	0.0957
Dipeptidyl peptidase III inhibitor	YL, YR, HL, LA, FL, PF	0.1043	PF	0.0217	PF	0.0217
CaMPDE inhibitor	IR	0.0043	IR	0.0043		
Renin inhibitor	IR, QF, LPL, LQL	0.0261	IR, QF	0.0087		
Xaa-Pro inhibitor	PL	0.0130	PL	0.0087	PL	0.0130
Lactocepin inhibitor	PL, LL, LP	0.0783	PL	0.0087	PL	0.0130
Inhibitor of tripeptidyl peptidase II	AAA, AA, AF, AP	0.0696				
Phospholipase A2 inhibitor	PY	0.0087				
Tubulin-tyrosine ligase inhibitor	AY	0.0174				
Inhibitor of cytosol alanyl aminopeptidase	AA, AAA, GGA, LL	0.0826				
Hypouricemic	LT, PT	0.0130				
D-Ala-D-Ala dipeptidase inhibitor	AA	0.0478				
**P06676**—Protein amino acid sequence: MATKIFSLLMLLALSACVANATIFPQCSQAPIASLLPPYLPSMIASVCENPALQPYRLQQAIAASNIPLSPLLF QQSPALSLVQSLVQTIRAQQLQQLVLPLINQVALANLSPYSQQQQFLPFNQLSTLNPAAYLQQQLLPFSQLATAYSQQQQLLPFNQLAALNPAAYLQQQILLPFSQLAAANRASFLTQQQLLPFYQQFAANPATLLQLQQLLPFVQLALTDPAASYQQHIIGGALF						
**Activity**	**Profiles of potential biological activity**		**GI: pepsin, trypsin, chymotrypsin**		**Subtilisin**	
	**Bioactive fragment**	**A**	**Bioactive fragment**	**A**	**Bioactive fragment**	**A**
ACE inhibitor	VLP, RL, IR, LPP, LVL, FP, IRAQQ, LAA, LSP, LQP, LNP, IRA, YL, LF, FY, FNQ, AY, FYQQ, LLP, PF, LPP LQQ, YSQQQQ, LNPA, PL, IA, IP, AP, LA, RA, AA, IF, IG, GA, GG, AI, SY, SF, LLF, LQ, LN, TQ, PP, PQ, ASL, LPF, LVQ, FQ, IL, ST, QP, LPL, LQL, LP	0.4625	PL, IF, PF	0.0083	RL, PL, PF	0.0125
Antibacterial peptide	AA	0.0333				
Immunomodulating	PFNQL, FLPFNQL	0.0125				
Stimulating (glucose uptake stimulating peptide)	VL, LV, IL, LI, II, LL	0.0708	VL	0.0042	VL	0.0042
Neuropeptide	YL, YR, PY, IL	0.0333			PY	0.0042
Regulating (phosphoglycerate kinase activity)	SL	0.0167	SL	0.0083		
Anti-inflammatory	PY, SACV, LLPF, ANP, LPF	0.0667			PY	0.0042
Hypotensive	AA, FY	0.0375				
Antioxidative	AY, IR, LLPF, LPL, LQL, LAN, YSQ, FY, YL, LT	0.0833				
Activating ubiquitin-mediated proteolysis	RA, LA	0.0333				
Alpha-glucosidase inhibitor	FY, PP, FY	0.0042				
Dipeptidyl peptidase IV inhibitor	PP, VA, MA, LA, FA, AP, PA, LP, LL, IP, SP, FP, GA, IA, RA, NP, TA, QP, FL, AL, SL, LPL, AA, PL, LQP, AS, AT, AY, DQ, FN, FQ, GG, HI, II, IL, IN, IR, KI, LI, LM, LN, LT, LV, MI, ML, NA, NL, NQ, NR, PF, PI, PQ, PS, PY, QA, QF, QH, QI, QL, QQ, QS, QT, QV, RL, SF, SV, SY, TD, TI, TK, TL, TQ, VL, VQ, YL, YQ, YR, YS, LPF	0.8417	AL, SL, PL, IN, PF, QL, VL	0.0667	AL, PL, ML, PF, OS, PY, QL, RL, TL, VL	0.0792
Dipeptidyl peptidase III inhibitor	YL, YR, LA, FA, FL, PF, SM	0.0833	PF	0.0250	PF	0.0250
CaMPDE inhibitor	IR	0.0042				
Renin inhibitor	IR, NR, QF, SF, LPL, LQL	0.0292				
Pancreatic lipase inhibitor	ASF	0.0042	ASF	0.0042		
Xaa-Pro inhibitor	PL	0.0125	PL	0.0042	PL	0.0083
Lactocepin inhibitor	PL, LL, LP	0.0917	PL	0.0042	PL	0.0083
Inhibitor of tripeptidyl peptidase II	VA, AAA, AA, AP	0.0500				
Phospholipase A2 inhibitor	PY	0.0125			PY	0.0042
Tubulin-tyrosine ligase inhibitor	AY	0.0125				
Inhibitor of cytosol alanyl aminopeptidase	AA, AAA, GGA, LL	0.0833				
Hypouricemic	LT, TL, ATL	0.0208			TL	0.0042
D-Ala-D-Ala dipeptidase inhibitor	AA	0.0333				
**P06678**—Protein amino acid sequence: MAAKIFALLALLALSANVATATIIPQCSQQYLSPVTAARFEYPTIQSYRLQQAIAASILRSLALTVQQPYALL QQPSLVNLYLQRIVAQQLQQQLLPTINQVVAANLDAYLQQQQFLPFNQLAGVNPAAYLQAQQLLPFNQLVRSPAAFLLQQQLLPFHLQVVANIAAFLQQQQLLPFYPQVVGNINAFLQQQQLLPFYPQDVANNVAFLQQQQLLPFSQLALTNPTTLLQQPTIGGAIF						
**Activity**	**Profiles of potential biological activity**		**GI: pepsin, trypsin, chymotrypsin**		**Subtilisin**	
	**Bioactive fragment**	**A**	**Bioactive fragment**	**A**	**Bioactive fragment**	**A**
Antiammnestic	LPPV	0.0038				
ACE inhibitor	RL, LPP, VAA, LAA, LNP, VAY, YL, LF, FNQ, AY, LLP, LQQ, PL, IA, LAP, IP, AF, AP, LA, VP, AA, GF, IF, VG, GA, AG, HL, MG, GG, AI, MNP, EV, LQ, LN, PT, TQ, AH, PP, PQ, VNP, LSW, AV, LPF, QP, LP, PF, LPP	0.4253	PL, AF, GF, AH, PF	0.0230	RL, VAY, PL, PP, VNP, PF	0.0192
Antibacterial peptide	AA	0.0192				
Immunomodulating	PFNQL, FLPPVT	0.0077				
Stimulating (vasoactive substance release)	SSS	0.0575				
Stimulating (glucose uptake stimulating peptide)	VL, LV, IV, II, LL	0.0575			VL	0.0038
Immunostimulating	GFL	0.0038				
Neuropeptide	YL, YR, PY	0.0268	PY	0.0038	PY	0.0038
Regulating (phosphoglycerate kinase activity)	GFL, SL	0.0038	SL	0.0038		
Anti-inflammatory	PY, LLPF, ANP, LPF, LSW	0.0192	PY	0.0038	PY	0.0038
Hypotensive	AA	0.0192				
Antioxidative	HL, AY, AH, TY, LLPF, SVL, LFV, LAN, LSW, QAY, YSQ, YL	0.0958	AH	0.0038		
Activating ubiquitin-mediated proteolysis	LA	0.0498				
HMG-CoA reductase inhibitor	IVG	0.0038				
Alpha-glucosidase inhibitor	PP	0.0038			PP	0.0038
Dipeptidyl peptidase IV inhibitor	PP, VA, LA, AP, PA, LP, VP, LL, VV, IP, SP, GA, IA, NP, QP, FL, HL, AL, SL, AA, PL, WQ, AF, AG, AH, AS, AT, AV, AY, EH, EV, FN, GF, GG, II, IN, IQ, LM, LN, LT, LV, MG, MN, MV, NA, NQ, NV, PF, PI, PN, PQ, PS, PT, PV, PY, QA, QF, QI, QL, QQ, QS, QT, RL, SV, SW, TI, TN, TQ, TS, TV, TY, VG, VL, VN, VQ, VS, VT, YL, YQ, YR, YS, LPF	0.8199	AL, SL, PL, AF, AH, EH, GF, PF, PY, QL, SW, VN	0.0690	PP, AL, PL, AS, PF, OS, PY, QF, QL, RL, VL, VS	0.0690
Dipeptidyl peptidase III inhibitor	YL, YR, GF, HL, LA, FL, PF, SM, GFL	0.1149	GF, PF	0.0077	PF	0.0038
Renin inhibitor	QF	0.0153			QF	0.0038
Pancreatic lipase inhibitor	SW	0.0038	SW	0.0038		
Xaa-Pro inhibitor	PL	0.0153	PL	0.0115	PL	0.0038
Lactocepin inhibitor	PL, LL, LP	0.0651	PL	0.0115	PL	0.0038
Inhibitor of tripeptidyl peptidase II	VA, GF, AA, AF, AP	0.0536	GF, AF	0.0077		
Phospholipase A2 inhibitor	PY	0.0038	PY	0.0038	PY	0.0038
Acylaminoacyl peptidase inhibitor	GF	0.0077	GF	0.0038		
Tubulin-tyrosine ligase inhibitor	AY	0.0230				
Glutamate carboxypeptidase inhibitor	FE	0.0038				
Inhibitor of cytosol alanyl aminopeptidase	AA, GGA, LL	0.0536				
Hypouricemic	IAT, LT, PT	0.0192				
D-Ala-D-Ala dipeptidase inhibitor	AA	0.0192				
**P04705**—Protein amino acid sequence: MAAKIFCLIMLLGLSASAATASIFPQCSQAPIASLLPPYLSPAMSSVCENPILLPYRIQQAIAAGILPLSPLFLQ QSSALLQQLPLVHLLAQNIRAQQLQQLVLANLAAYSQQQQLPLVHLLAQNIRAQQLQQLVLANLAAYSQQQQFLPFNQQLAAAYPRQFLPFNQLAALNSHAYVQQ						
**Activity**	**Profiles of potential biological activity**		**GI: pepsin, trypsin, chymotrypsin**		**Subtilisin**	
	**Bioactive fragment**	**A**	**Bioactive fragment**	**A**	**Bioactive fragment**	**A**
ACE inhibitor	PF, LPP, ILP, IR, LPP, LVL, FP, IRAQQ, YPR, PR, LAA, LSP, IRA, YL, LF, FNQ, AY, LSPA, YP, LLP, LQQ, YSQQQQ, HLL, PL, IA, AP, LA, RA, AA, IF, GI, GL, AG, HL, AI, LG, LQ, LN, PP, PQ, ASL, LPF, YV, IL, LPL, LP, GIL	0.5389	IR, PR, AY, PL, IF, GL, PF	0.0500	PL, IF, GL, PF	0.0333
Antibacterial peptide	AA	0.0444				
Immunomodulating	PFNQL, FLPFNQL	0.0111				
Stimulating (glucose uptake stimulating peptide)	VL, LV, IL, LI, LL	0.0833	VL	0.0111	VL	0.0111
Neuropeptide	YL, YR, PY, IL	0.0333	PY	0.0056	PY	0.0056
Regulating (phosphoglycerate kinase activity)	SL	0.0056				
Anti-inflammatory	PY, LPF	0.0222	PY	0.0056	PY	0.0056
Hypotensive	AA	0.0444				
Antioxidative	HL, AY, IR, FC, LPL, LAN, YSQ, YL	0.0944	AY, IR	0.0167		
Activating ubiquitin-mediated proteolysis	RA, LA	0.0556				
Alpha-glucosidase inhibitor	YP, PP	0.0111				
Dipeptidyl peptidase IV inhibitor	PP, MA, LA, AP, PA, LP, LL, HA, SP, FP, YP, IA, RA, NP, TA, FL, HL, AL, SL, GL, LPL, AA, PL, AG, AS, AT, AY, FN, GI, IL, IM, IQ, IR, KI, LI, LN, LV, ML, NL, NQ, PF, PI, PQ, PY, QA, QF, QL, QN, QQ, QS, RI, SH, SI, SV, VH, VL, VQ, YL, YR, YS, YV, LPF	0.7944	GL, PL, AY, IM, IR, PF, PY, QF, QL, SH, VH, VL	0.1000	AL, GL, PL, AS, PF, PY, VL	0.0667
Dipeptidyl peptidase III inhibitor	YL, YR, PR, HL, LA, FL, PF	0.1000	PR, PF	0.0167	PF	0.0111
CaMPDE inhibitor	IR	0.0111	IR	0.0111		
Renin inhibitor	IR, QF, LPL	0.0389	IR, QF	0.0167		
Xaa-Pro inhibitor	PL	0.0222	PL	0.0167	PL	0.0222
Lactocepin inhibitor	PL, LL, LP	0.0944	PL	0.0167	PL	0.0222
Inhibitor of tripeptidyl peptidase II	AAA, AA, AP	0.0556				
Phospholipase A2 inhibitor	PY	0.0111	PY	0.0056	PY	0.0056
Tubulin-tyrosine ligase inhibitor	AY	0.0222	AY	0.0056		
Inhibitor of cytosol alanyl aminopeptidase	AA, AAA, LL	0.0833				
D-Ala-D-Ala dipeptidase inhibitor	AA	0.0444				

Protein amino acid sequence obtained from UniProtKB database. The profile of potential bioactive fragments was obtained at the BIOPEP-UWM database by selecting Profile of Potential Biological Activity, For your Sequence. The other bioactive fragments were obtained at the BIOPEP-UWM database by selecting Bioactive Peptide and choosing the enzyme. A: frequency of bioactive occurrence; GI: gastrointestinal digestion. A, Alanine; R, Arginine; N, Asparagine; D, Aspartic acid; C, Cysteine; Q, Glutamine; E, Glutamic acid; G, Glycine; H, Histidine; I, Isoleucine; L, Leucine; K, Lysine; M, Methionine; F, Phenylalanine; P, Proline; S, Serine; T, Threonine; W, Tryptophan; Y, Tyrosine; V, Valine.

**Table 3:** Predicted potential for obtaining bioactive peptides for α-Zein with 22 kDa.

α-Zein—22 kDa						
**P06679**—Protein amino acid sequence: LALLALLALFVSATNAFIIPQCSLAPSAIIPQFLPPVTSMGFEHLAVQAYRLQQALAASVLQQPINQLQQQSL AHLTIQTIATQQQQQFLPSVSQLDVVNPVAYLQQQLLASNPLALANVAAYQQQQQLQQFLPALSQLAMVNPAAYLQQQQLLSSSPLAVGNAPTYLQQQLLQQIVPALTQLAVANPAAYLQQLLPFNQLTVSNSAAYLQQRQQLLNPLEVPNPLVAGFLQQQQLLPYSQFSLMNPALSWQQPIVGGAIF						
	**Profiles of potential biological activity**		**GI: pepsin, trypsin, chymotrypsin**		**Subtilisin**	
**Activity**	**Bioactive fragment**	**A**	**Bioactive fragment**	**A**	**Bioactive fragment**	**A**
Alpha-amylase inhibitor	FY	0.0083				
ACE inhibitor	ARF, PF, RL, LY, RF, VAA, LVR, LSP, YL, FY, FNQ, AY, YP, LLP, LQQ, IA, IP, AF, LA, YA, AA, IF, VG, IG, GA, AG, HL, DA, GV, GG, AI, VR, TNP, SY, AR, EY, FAL, LQ, PT, PQ, VAF, VRSP, AFLL, VNP, LPF, AFL, IL, LR, QP, LP, AYLQAQQLLPFNQLVRSPAA	0.4917	AF, IF, VR, EY, VAF, PF	0.0208	RL, AG, HL, EY, PQ, VAF, IL, PF	0.0292
Celiac toxic	QQPY	0.0042				
Antibacterial peptide	AA	0.0292				
Immunomodulating	PFNQL, FLPFNQL, SQLALTNPT, PFNQLAG	0.0208				
Stimulating (glucose uptake stimulating peptide)	LV, IV, IL, II, LL	0.0667			IL	0.0042
Neuropeptide	YL, YR, PY, IL	0.0292			IL	0.0042
Regulating (phosphoglycerate kinase activity)	SL	0.0083	SL	0.0042		
Anti-inflammatory	PY, LLPF, LPF	0.0500				
Hypotensive	AA, FY	0.0375				
Antioxidative	HL, AY, LY, LLPF, AYLQAQQLLPFNQLVRSPAA, YPQ, YA, FY, YL, LT	0.0875			HL	0.0042
Alpha-glucosidase inhibitor	YP, LR, FY					
Activating ubiquitin-mediated proteolysis	LA	0.0208				
Dipeptidyl peptidase IV inhibitor	VA, MA, LA, FA, PA, LP, LL, VV, IP, SP, YP, GA, IA, NP, TA, QP, FL, HL, AL, SL, VR, AA, AF, AG, AS, AT, AY, EY, FN, GG, GV, II, IL, IN, IQ, KI, LT, LV, NA, NL, NN, NQ, NV, PF, PQ, PS, PT, PV, PY, QA, QD, QF, QL, QQ, QS, QV, QY, RI, RL, SI, SY, TI, TL, TN, TT, TV, VG, VN, VQ, VT, YA, YL, YR, LPF	0.8125	AL, SL, VR, AF, EY, IN, PF, QL, TN, VN	0.0875	HL, AL, AG, EY, IL, PF, PQ, QL, RL	0.0792
Dipeptidyl peptidase III inhibitor	LR, YL, YR, RF, DA, HL, LA, FA, FL, PF	0.1083	PF	0.0250	HL, PF	0.0292
Renin inhibitor	LR, QF, YA, LY	0.0167				
Leucyltransferase inhibitor	RF	0.0042				
Lactocepin inhibitor	LL, LP	0.0750				
Inhibitor of tripeptidyl peptidase II	VA, AA, AF	0.0708	AF	0.0042		
Phospholipase A2 inhibitor	PY	0.0042				
Tubulin-tyrosine ligase inhibitor	EY, AY, YA	0.0167	EY	0.0042	EY	0.0042
Alanine carboxypeptidase inhibitor	YA	0.0042				
Glutamate carboxypeptidase inhibitor	FE, DA	0.0042				
Inhibitor of cytosol alanyl aminopeptidase	AA, GGA, LL	0.0792				
Hypouricemic	FH, LR, LPT, LT, PT, TL, TT	0.0458				
D-Ala-D-Ala dipeptidase inhibitor	AA	0.0292				
**Q94IM1**—Protein amino acid sequence: MATKILSLLALLALFASATNASIIPQCSLAPSSIIPQFLPPVTSMAFEHPAVQAYRLQQAIAASVLQQPIAQL QQQSLAHLTIQTIATQQQQQFLPALSHLAMVNPIAYLQQQLLASNPLGLANVVANQQQQQLQQFLPALSQLAMVNPAAYLQQQQLLSSSPLAVANAPTYLQQELLQQIVPALTQLAVANPVAYLQQLLPFNQLTMSNSVAYLQQRQQLLNPLAVANPLVAAFLQQQQLLPYNRFSLMNPVLSRQQPIVGGAIF						
	**Profiles of potential biological activity**		**GI: pepsin, trypsin, chymotrypsin**		**Subtilisin**	
**Activity**	**Bioactive fragment**	**A**	**Bioactive fragment**	**A**	**Bioactive fragment**	**A**
Antiammnestic	LPPV	0.0038				
ACE inhibitor	RL, LPP, RF, VAA, LNP, VAY, YL, LF, FNQ, AY, LLP, LQQ, PLG, PL, IA, LAP, IP, AF, AP, LA, VP, AA, IF, VG, GA, GL, HL, GG, AI, LG, MNP, LQ, LN, PT, TQ, AH, PP, TQ, AH, PP, PQ, HP, VNP, AV, LPF, AFL, IAQ, AQL, IAY, IL, YN, QP, LP	0.4286	PL, AF, GL, AH, IL, PF	0.0263	RL, VAY, PL, GL, HL, MNP, PP, PF	0.0301
Antibacterial peptide	AA	0.0113				
Immunomodulating	PFNQL, FLPPVT	0.0075				
Opioid	PLG	0.0038				
Stimulating (vasoactive substance release)	SSS	0.0639				
Stimulating (glucose uptake stimulating peptide)	VL, LV, IV, IL, II, LL	0.0639	IL	0.0038	VL	0.0075
Neuropeptide	YL, YR, PY, IL	0.0301	PY, IL	0.0075	PY	0.0038
Regulating (phosphoglycerate kinase activity)	SL	0.0150	SL	0.0038		
Anti-inflammatory	PY, LLPF, ANP, LPF	0.0188	PY	0.0038	PY	0.0038
Hypotensive	AA	0.0113				
Antioxidative	HL, AY, AH, EL, TY, LLPF, SVL, LAN, QAY, IAY, YL, LT	0.0865	AH	0.0038	HL	0.0038
Activating ubiquitin-mediated proteolysis	LA	0.0414				
HMG-CoA reductase inhibitor	IVG	0.0038				
Alpha-glucosidase inhibitor	PP	0.0038			PP	0.0038
Dipeptidyl peptidase IV inhibitor	PP, VA, MA, LA, FA, AP, PA, LP, VP, LL, VV, IP, SP, HP, GA, IA, NP, QP, FL, HL, AL, SL, GL, AA, PL, AF, AH, AS, AT, AV, AY, EH, FN, GG, II, IL, IQ, KI, LM, LN, LT, LV, MN, MV, NA, NQ, NR, NV, PF, PI, PQ, PS, PT, PV, PY, QA, QE, QF, QI, QL, QQ, QS, QT, RL, SH, SI, SV, TI, TK, TM, TN, TQ, TS, TY, VG, VL, VN, VQ, VT, YL, YN, YR, LPF	0.8083	AL, SL, GL, PL, AF, AH, EH, IL, PF, PY, QL, SH, TM, VN,	0.0714	PP, HL, AL, GL, PL, AS, PF, PY, QL, RL, VL	0.0526
Dipeptidyl peptidase III inhibitor	YL, YR, RF, HL, HP, LA, FA, FL, PF, SM	0.1053	PF	0.0038	HL, PF	0.0075
Leucyltransferase inhibitor	RF	0.0038				
Xaa-Pro inhibitor	PL	0.0150	PL	0.0113	PL	0.0038
Lactocepin inhibitor	PL, LL, LP	0.0639	PL	0.0113	PL	0.0038
Inhibitor of tripeptidyl peptidase II	VA, AA, AF, AP	0.0526	AF	0.0038		
Phospholipase A2 inhibitor	PY	0.0038	PY	0.0038	PY	0.0038
Tubulin-tyrosine ligase inhibitor	AY	0.0188				
Glutamate carboxypeptidase inhibitor	FE	0.0038				
Inhibitor of cytosol alanyl aminopeptidase	AA, GGA, LL	0.0451				
Hypouricemic	IAT, LT, PT	0.0188				
D-Ala-D-Ala dipeptidase inhibitor	AA	0.0113				

Protein amino acid sequence obtained from UniProtKB database. The profile of potential bioactive fragments was obtained at the BIOPEP-UWM database by selecting Profile of Potential Biological Activity, For your Sequence. The other bioactive fragments were obtained at the BIOPEP-UWM database by selecting Bioactive Peptide and choosing the enzyme. A: frequency of bioactive occurrence; GI: gastrointestinal digestion. A, Alanine; R, Arginine; N, Asparagine; D, Aspartic acid; C, Cysteine; Q, Glutamine; E, Glutamic acid; G, Glycine; H, Histidine; I, Isoleucine; L, Leucine; K, Lysine; M, Methionine; F, Phenylalanine; P, Proline; S, Serine; T, Threonine; W, Tryptophan; Y, Tyrosine; V, Valine.

**Table 4:** Scores at PeptideRanker of the bioactive peptide fragments with biological potential obtained from BIOPEP-UWM and peptide sequences with the same bioactive fragment identified in scientific studies in the literature.

Score ^a^	Bioactive Fragment	Biological Activity	Corn Specie	Peptide Amino Acid Sequence ^b^	Reference
**0.9978**	FCF	ACE inhibitor			
**0.9964**	CF	ACE inhibitor			
**0.9947**	GF	ACE and Renin inhibitor, and DPP-III and DPP-IV	Corn silk; corn	C**GF**PPAGYLRR; YG**GF**M	[52,69]
**0.9939**	FP	ACE inhibitor and DPP-IV			
**0.9934**	PF	ACE inhibitor and DPP-IV	Corn	L**PF**	[61]
**0.9869**	LF	ACE inhibitor	α-zein	**LF**	[79]
**0.9865**	RF	ACE inhibitor and DPP-III and DPP-IV	Corn	AARP**RF**NH_2_	[80]
**0.9824**	FY	ACE and alpha-amylase inhibitor, hypotensive and antioxidative			
**0.9749**	LPF	ACE inhibitor, anti-inflammatory, and DPP-IV	α-zein	FNQLAALNSAAYLQQQQL**LPF**SQLA; QLADVSPAAFLTQQQL**LPF**YLHAM; AYLQAQQL**LPF**NQLVRSPAA; CFFNQLAALNSAAYLQQQQL**LPF**SQLA; CFAYLQAQQL**LPF**NQLVRSPAA; CFQLADVSPAAFLTQQQL**LPF**YLHAM	[16]
**0.9732**	AF	ACE inhibitor, Inhibitor of tripeptidyl peptidase II, and DPP-IV	α-zein	**AF**, QLADVSPA**AF**LTQQQLLPFYLHAM, CFQLADVSPA**AF**LTQQQLLPFYLHAM	[16,79]
**0.9491**	IF	ACE inhibitor and DPP-IV	Corn	T**IF**PQ	[67]
**0.9487**	SF	ACE and renin inhibitor and DPP-IV			
**0.9439**	MG	ACE inhibitor and DPP-IV			
**0.9389**	LLF	ACE inhibitor	Corn	**LLF**	[61]
**0.9298**	ARF	ACE inhibitor			
**0.9285**	AFL	ACE inhibitor	α-zein	QLADVSPA**AFL**TQQQLLPFYLHAM, CFQLADVSPA**AFL**TQQQLLPFYLHAM	[16,79]
**0.9160**	FQ	ACE inhibitor and DPP-IV	Corn	**FQ**	[61]
**0.8902**	FAL	ACE inhibitor			
**0.8892**	AFLL	ACE inhibitor			
**0.8873**	GG	ACE inhibitor and DPP-IV			
**0.8869**	PP	ACE and alpha-glucosidase inhibitor and DPP-IV	γ-zein and α-zein	VHLP**PP**; OGL**PP**G**PP**I**PP**; SLL**PP**YLSPA; L**PP**	[10,81]
**0.8282**	LSW	ACE inhibitor, anti-inflammatory, and antioxidative			
**0.8280**	LPP	ACE inhibitor and DPP-IV	α-zein, corn gluten meal	VH**LPP**P	[79,81,82].
**0.8111**	PL	ACE inhibitor and DPP-IV	α-zein; Zein	PPIPPGP**PL**OG; V**PL**	[83,84]
**0.8087**	GL	ACE, Xaa-Pro, and lactocepin inhibitor, and DPP-IV			
**0.8013**	PLG	ACE inhibitor and DPP-IV			
**0.9802**	GFL	Regulating (phosphoglycerate kinase activity) and DPP-III	Corn	G**GFL**	[52]
**0.9960**	FC	Antioxidant			
**0.9464**	LLPF	Antioxidant and anti-inflammatory	corn gluten meal; α-zein	**LLPF**; QQ**LLPF**, QQI**LLPF**, QI**LLPF**; Q**LLPF**	[9,67,85]
**0.9934**	PF	DPP-IV and DPP-III			
**0.9896**	FL	DPP-IV and DPP-III			
**0.9558**	FA	DPP-IV and DPP-III			
**0.9512**	FN	DPP-IV			
**0.9461**	QF	DPP-IV and renin inhibitor			
**0.9339**	SW	DPP-IV and pancreatic lipase inhibitor			
**0.9285**	AFL	DPP-IV			
**0.8945**	ML	DPP-IV			

^a^ It presented only the fragments with a score above 0.8; ^b^ Bold: bioactive fragment. A, Alanine; R, Arginine; N, Asparagine; D, Aspartic acid; C, Cysteine; Q, Glutamine; E, Glutamic acid; G, Glycine; H, Histidine; I, Isoleucine; L, Leucine; K, Lysine; M, Methionine; F, Phenylalanine; P, Proline; S, Serine; T, Threonine; W, Tryptophan; Y, Tyrosine; V, Valine.

## Data Availability

Data derived from public domain resources. The data presented in this study are available in UniProtKB at https://www.uniprot.org/uniprotkb (accessed on 8 August 2024), referenced by the code of protein mentioned in the text.
